# A case report of epithelial adenomatoid hamartoma misdiagnosed as a nasal poly

**DOI:** 10.1097/MD.0000000000041424

**Published:** 2025-02-14

**Authors:** Siyu Liu, Bin Bai, Yu Feng, Maocai Li, Lianqing Li, Lili Gong

**Affiliations:** a Department of Otorhinolaryngology Head and Neck Surgery, Liaocheng People’s Hospital, Liaocheng, China; b School of Clinical Medicine, Shandong Second Medical University, Weifang, China; c Department of pathology, Liaocheng City Second People’s Hospital, Linqing, China; d Shandong First Medical University (Shandong Academy of Medical Sciences), Jinan, China.

**Keywords:** nasal mass, nasal polyps, respiratory epithelial adenomatoid hamartoma

## Abstract

**Rationale:**

Respiratory epithelial adenomatoid hamartomas (REAH) are benign tumors. The clinical presentation of REAH often resembles that of nasal polyps or inverted papillomas. Owing to the lack of specific clinical symptoms or imaging findings, the diagnosis is usually confirmed only by pathological examination after surgery and misdiagnosis is common. Therefore, we report this case of nasal REAH that was misdiagnosed as a nasal polyp. Clinical cases of nasal polyps with hamartoma are very rare. Pathological biopsy, complete removal of the lesion by surgery and postoperative follow-up are essential for the diagnosis and treatment of REAH.

**Patient concerns:**

The patient had been experiencing bilateral anosmia for 6 years without any obvious triggers. He also experienced nasal congestion and mucopurulent nasal discharge after exposure to cold and alcohol.

**Diagnosis:**

Analyzing the pathological findings that the patient was diagnosed with right-sided respiratory epithelial adenomatoid pleomorphic histiocytoma and left-sided nasal polyps.

**Interventions:**

Nasal endoscopic surgery was performed to remove bilateral nasal lesions and send them for routine pathological examination.

**Outcomes:**

After surgery, the patient’s sense of smell was slightly restored to normal.

**Lessons:**

Our case report findings suggest that clinical attention should be paid to the possibility of nasal REAH with nasal polyps in bilateral nasal polypoid masses, particularly if the olfactory fissure is involved.

## 1. Introduction

A misshapen tumor is a tumor-like mixture of malformed intrinsic tissues with excess of >1 cellular components in a particular part of the body.^[1]^ Misshapen tumors occur at a wide range of sites, and the main origins are the skin or subcutaneous tissues, as well as visceral organs; the head and neck are not commonly involved. Hamartomas are classified as epithelial, mesenchymal, or mixed epithelial-mesenchymal. Respiratory epithelial adenomatoid pleomorphic histiocytoma (REAH) is a subtype of epithelial pleomorphic histiocytoma and the most common type of pleomorphic histiocytoma in the nasal cavity and nasopharynx.^[2]^ Herein, we report a case of nasal REAH that was misdiagnosed as a nasal polyp. After analyzing the pathological findings, the patient was diagnosed with a right-sided REAH and left-sided nasal polyps. This condition is extremely rare in clinical practice and hence needs to be reported to aid clinicians in decision-making regarding diagnosis and treatment.

## 2. Case report

**Figure 1.**
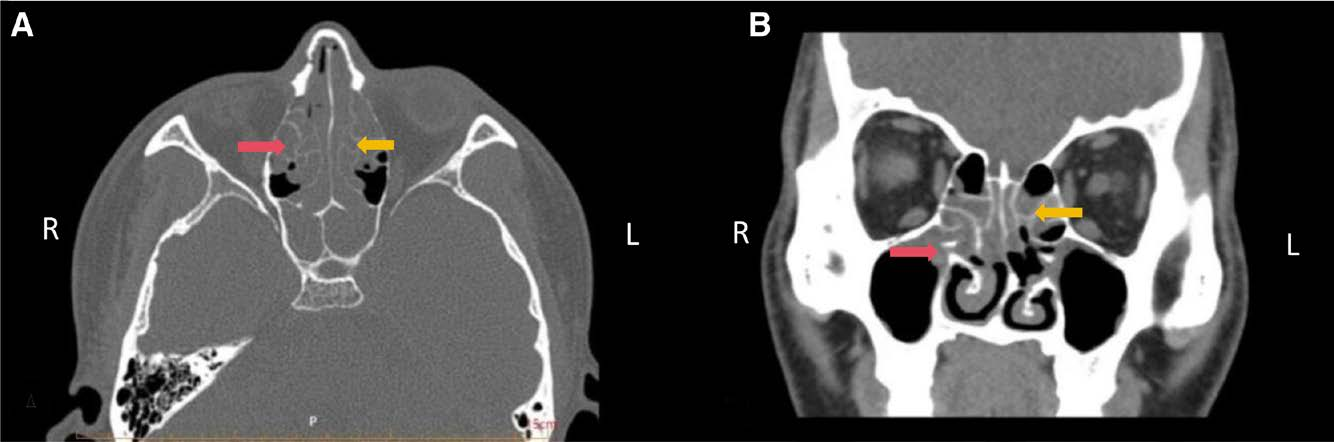
(A) Axial CT of the nasal cavity. (B) Coronal CT of the nasal cavity. Yellow scissors indicate a nasal polyp in the low-density area of the left nasal cavity; red scissors indicate REAH in the left nasal cavity with low density. CT = computed tomography, REAH = respiratory epithelial adenomatoid hamartomas.

The patient was a 35-year-old man who had been experiencing bilateral anosmia for 6 years without any obvious triggers. He also experienced nasal congestion and mucopurulent nasal discharge after exposure to cold and alcohol. The main symptom was hypo-olfactory, and he had no previous history of asthma or sinus surgery. Nasal endoscopy revealed bilateral nasal polypoid growth in the middle nasal passage and bilateral enlargement of the inferior turbinate. Sinus computed tomography (CT) revealed abnormal low-density shadows in the middle nasal passages bilaterally and anterior deviation of the nasal septum to the left (Fig. 1).

**Figure 2.**
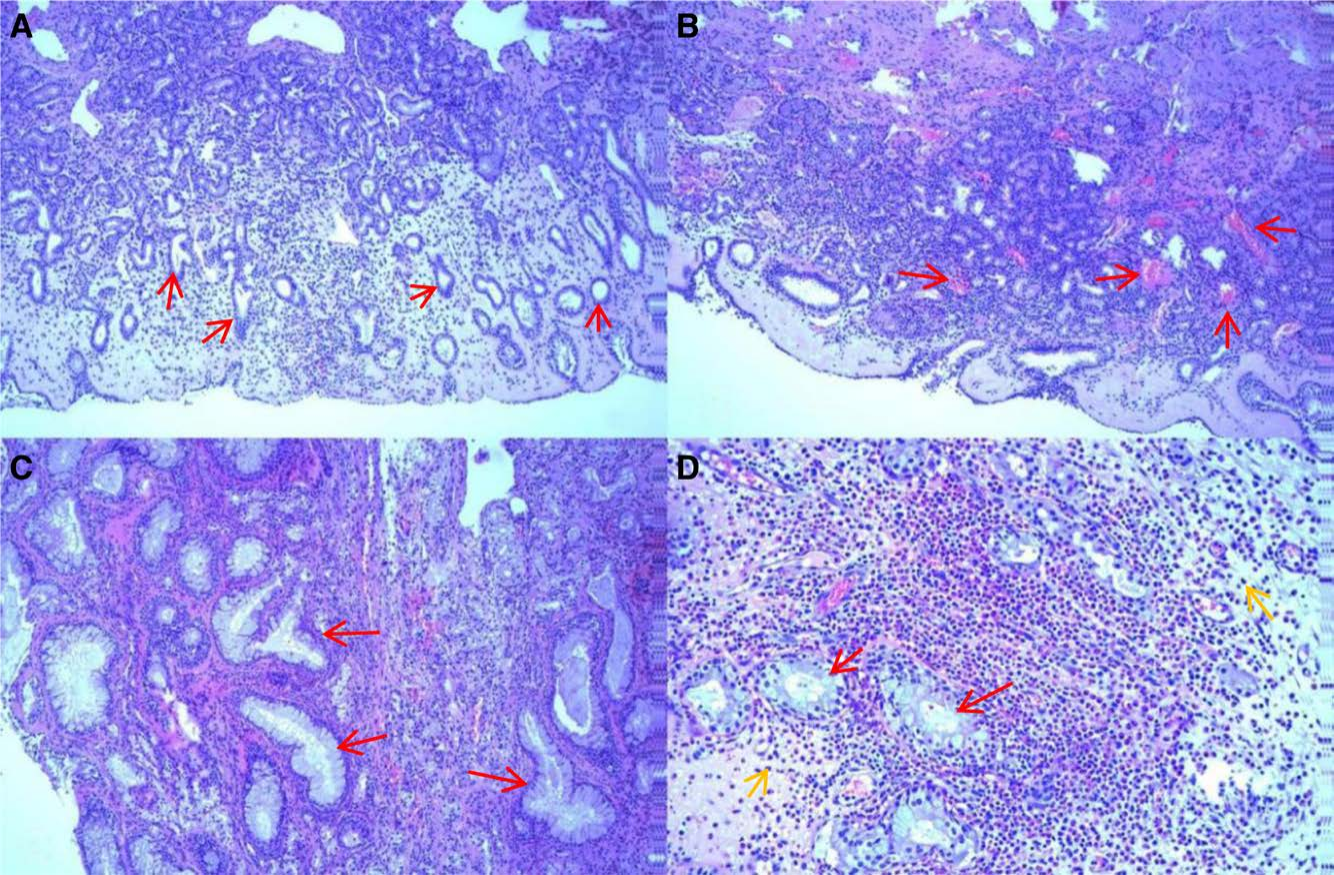
Results of the pathologic examination of the resected tumor. (A) At 10 × 10 magnification, predominantly massive plasma glandular hyperplasia is noted (red arrow), forming an adenomatous structure with scattered phagocytic infiltration. (B) Interstitial vascular proliferation is evident (red arrow). (C) At 10 × 10 magnification, predominantly massive mucous gland hyperplasia is noted (red arrow), forming adenomatous structures with scattered eosinophilic and centrocytic infiltrate. (D) At 10 × 20 magnification, diffuse infiltration of eosinophils (yellow arrow) with residual mucous glands (red arrow) is noted.

The patient underwent endoscopic resection of the nasal lesions under general anesthesia; during the procedure, it was found that the bilateral nasal polypoid masses were located in the middle nasal passages and olfactory fissure. Root tips were located in the hook, middle turbinate, sieve bubble, and upper turbinate. Polypoid tissue was excised and sent for routine pathological examination. The results showed that the right nasal mass had a large number of proliferating plasma and mucous glands, forming an adenomatous structure with scattered infiltration of phagocytic and neutrophilic granulocytes and marked interstitial vascular hyperplasia (Fig. 2). The left nasal mass showed microscopic interstitial edema and mixed inflammatory cell infiltration with predominant eosinophils. Based on the pathological findings, the patient was diagnosed with REAH of the right nasal cavity and a nasal polyp on the left side. After surgery, the patient’s sense of smell was slightly restored to normal.

## 3. Discussion

REAH is the most common type of nasal pleomorphic histiocytoma. It originates from the surface epithelium and is characterized by the overproliferation of the normal glandular component of the respiratory epithelium.^[3]^ The average age at onset of nasal REAH is 50 to 60 years, with a male predominance. The pathogenesis of the disease is still unclear, and some studies have shown that the onset of REAH is closely related to long-term nasal polyposis, repeated sinus surgery, and asthma, which mostly presents as an isolated lesion, but can also be accompanied by nasal polyps, inverted papilloma, or malignant tumors.^[4]^ The clinical symptoms of REAH vary and mainly include a decreased sense of smell, nasal congestion, nasal leakage, rhinorrhea, headache, and facial pressure.^[5]^

The patient we reported was diagnosed with rare REAH accompanied by nasal polyps, the main symptom was hypo-olfactory symptoms, and he had no previous history of asthma or sinus surgery. Bilateral nasal polyps were considered in the initial diagnosis, and the possibility of unilateral REAH was ignored.

REAH is a benign tumor with a slow growth rate, is mostly solitary, and is characterized on CT by an olfactory fissure that is >10 mm wide and generally does not invade bony structures.^[6,7]^ The histological manifestation of REAH is glandular hyperplasia, and the hyperplastic glands are composed of a stratified ciliated respiratory epithelium mixed with mucous cells. One of the main features of glands is that they expand with mucus and are separated by mesenchyma. The surface epithelium descended into the submucosa and was directly connected to the gland,^[8]^ which was consistent with the pathological smear of this patient. The clinical symptoms of nasal REAH are not specific, and it is usually necessary to differentiate it from benign inflammatory polyps and inverted papillomas on imaging and pathology.

Benign inflammatory polyp have a translucent, lytic, flesh-like appearance and bilateral lesions; they mainly occur in the area of sinus drainage and rarely in the olfactory fissure alone.^[9]^ In this case, the lack of specific imaging findings further complicates clinical diagnosis. However, for clinicians who encounter CT findings of swelling and widening of the olfactory fissure, this can serve as a differential diagnosis for nasal REAH and nasal polyps. Inverted papilloma (mulberry papilloma), which is often located in the nasal vestibule and septum, is locally invasive and can cause bone thinning and curvature. CT examination shows that the origin of the varus papilloma may be focal osteosclerosis, which can be used as the basis for differentiating it from REAH.^[10]^ In this case, CT showed no bone changes, and an inverted papilloma was not considered.

Surgical treatment with intraoperative pathological biopsy remains the most effective method for confirming the diagnosis of nasal histiocytoma. Recurrence of nasal dissecting tumor after surgery is uncommon, and the disease is rarely malignant. However, nasal polyps have a high recurrence rate after endoscopic surgical resection;^[11]^ therefore, in this case, the mucosa of the root tip where the lesion was located was complete as possible during the surgery, while avoiding unnecessary bone resection and excessive damage to the mucosa. Patients should be actively followed-up after surgery to observe whether the nasal polyp recurs and the recovery of olfaction. For this case, there was no recurrence of nasal polyps and hamartoma in the short-term follow-up after operation, and the olfactory function was significantly improved.

## 4. Conclusions

Nasal REAH is extremely rare in clinical practice and has a high rate of misdiagnosis, because it is difficult to diagnose it preoperatively. Pathological biopsy is the only means of confirming REAH, and clinical attention should be paid to the possibility of nasal REAH with nasal polyps in bilateral nasal polypoid masses, particularly if the olfactory fissure is involved. Feasible preoperative biopsy diagnosis when necessary, further making surgical diagnosis and treatment plan. Aggressive preoperative refinement of all tests and complete intraoperative resection are effective treatments for respiratory epithelial adenomatoid misshapen tumors; however, patients should be monitored for the recurrence of nasal polyps.

## Acknowledgments

I sincerely thank my tutor Li Lianqing for his valuable advice and careful guidance on the revision of the article, and thank all the authors for their selfless help in data collection.

## Author contributions

**Data curation:** Lianqing Li, Bin Bai.

**Writing – original draft:** Lianqing Li, Siyu Liu, Maocai Li.

**Writing – review & editing:** Lianqing Li, Yu Feng, Maocai Li, Lili Gong.
